# Identification of epithelial, mesenchymal, and platelet-associated circulating tumour cells with translational implications in oral squamous cell carcinoma

**DOI:** 10.1038/s41598-026-57427-z

**Published:** 2026-06-10

**Authors:** Geeta S. Boora, Anshika Chauhan, Rijuneeta Gupta, Jaimanti Bakshi, Sushmita Ghoshal, Arnab Pal

**Affiliations:** 1 Department of Biochemistry, Post Graduate Institute of Medical Education and Research, Chandigarh, India; 2 Department of Otolaryngology and Head Neck Surgery, Post Graduate Institute of Medical Education and Research, Chandigarh, India; 3 Department of Radiotherapy and Oncology, Post Graduate Institute of Medical Education and Research, Chandigarh, India

**Keywords:** Circulating Tumour Cells, Oral Squamous Cell Carcinoma, Fluorescence-Activated Cell Sorting, CTC Enumeration, CTC-platelets heterotypic clusters, Epithelial to Mesenchymal Transition, Platelet Exclusion, Limit of Detection, Limit of Quantification, Biomarkers, Cancer, Oncology

## Abstract

Metastatic dissemination is driven by rare Circulating Tumour Cells (CTCs) that exhibit pronounced phenotypic plasticity and dynamic interactions with different components in the bloodstream. In Oral Squamous Cell Carcinoma (OSCC), limited resolution of epithelial, mesenchymal, and platelet-associated CTC-states has constrained biological understanding of early tumour spread and hindered translational interpretation. In this study, we identify, count and characterize distinct epithelial, mesenchymal, and platelet-associated CTC-states in a cohort of 33 patients using a strategic workflow involving hematopoietic cell depletion with multiparametric flow-cytometric analysis. The FACS (Fluorescence-assisted cell sorting)-strategy incorporated epithelial tumour-associated markers (EpCAM, EGFR, and Cytokeratin), mesenchymal markers (VIM and N-Cadherin), and platelet/leukocyte markers (CD41 and CD45) to resolve biologically distinct CTC-subpopulations. Analytical robustness was established through spike-in experiments using OSCC cell line i.e., Cal27, demonstrating linear detection across clinically relevant ranges(1-100cells/mL of blood, Spearman’s *r* = 0.8738, *p* < 0.0001) and sensitive recovery of rare tumour cells with a Limit of Detection of 1 cell/mL, Limit of Quantification of 10 cells/mL, and Limit of Blank of 1.779 cells/mL. Molecular validation of sorted populations using whole transcriptome amplification-quantitative PCR confirmed tumour-associated marker expression and absence of hematopoietic contamination. Application of this approach in OSCC patients revealed detectable CTCs in 70% of the cases (ranging from 14.20 to 340.5 CTCs-related events/mL). Importantly, CTCs were identified across discrete phenotypic states, including platelet-associated CTC-clusters that were not captured by epithelial marker-restricted strategies. Quantitative comparison of CTC subtype proportions revealed majority (nearly 83%) of CTCs present as platelet clusters, followed by Epithelial-Single CTCs (21.21%). In comparison, CTCs with only mesenchymal phenotype constitute only 3.22% of the total CTC-population. Receiver operating characteristic analysis supported the discriminatory capacity of CTC enumeration and enabled the definition of a biologically relevant threshold of > 19.40 events mL for CTC positivity. Taken together, our findings outline a practical workflow for identifying and quantifying different CTC-states in OSCC, with platelet-associated CTCs emerging as the predominant population detected. These findings support the importance of incorporating phenotypic heterogeneity and platelet-associated tumour cell interactions into future liquid biopsy-based translational pathology research and clinical biomarker development in OSCC.

**Supplementary Information:**

The online version contains supplementary material available at 10.1038/s41598-026-57427-z.

## Introduction

Metastasis remains the major cause of cancer-related mortality and is typically detectable clinically or by radiological imaging and/or confirmed by tissue biopsy^1^. However, the metastatic dissemination begins much before the establishment of clinically detectable metastasis and is driven by Circulating Tumour Cells (CTCs), which shed from primary tumour into the circulation^2^. These cells represent a dynamic and biologically active intermediate state of tumour progression. Thus, early detection and characterisation of CTCs in blood through liquid biopsy approaches provide a minimally invasive approach for real-time and longitudinal monitoring of tumour spread^3^.

Beyond enumeration, growing evidence indicates that CTCs exist in multiple phenotypic and functional states that influence their metastatic potential. CTCs represent a dynamic intermediate state undergoing Epithelial-Mesenchymal Transition (EMT) at the time of intravasation and undergoing Mesenchymal-Epithelial Transition (MET) at the time of extravasation and metastatic colonization^4,5^. This phenotypic plasticity results in heterogeneous CTC populations expressing epithelial, mesenchymal, or hybrid marker profiles.

Additionally, CTCs often interact with various blood components like neutrophils, monocytes and platelets to form heterotypic clusters^6–9^. Among these, CTC-platelet clusters are of particular biological relevance^10^. As platelets physically shield these clusters from immune attack and shear forces in the circulation, they enhance their survival in circulation and promote metastatic seeding^11^.

Despite their high clinical importance, the analysis of CTCs remains technically challenging due to their extreme rarity, formation of heterotypic clusters and phenotypic heterogeneity. The currently available CTC enumeration/isolation methods based on physical properties, such as size-based filtration or microfluidic devices, pose limitations related to purity and underestimation of CTC load. The size of CTCs may range from 4 μm to 30 μm, even within the same patient^12^. Thus, a smaller filter cutoff may capture non-tumour cells of similar sizes, compromising purity, whereas large filter cutoffs may lose small-sized CTCs, leading to underestimation of CTC burden^13,14^. The size-based filtration is optimally suited for large CTCs and their heterotypic cluster with cells like neutrophils and monocytes. The marker-based approaches also possess significant limitations due to the dynamic expression of surface markers during different stages of EMT and MET. Currently, the FDA-approved CellSearch™ system only detects CTCs of epithelial phenotype, i.e. Epithelial Cell Adhesion Molecule (EpCAM)-positive Cytokeratin (CK)-positive, and CD45-negative, and misses CTCs undergoing EMT^15,16^. Similarly, Parsortix is a label-independent microfluidic platform that enriches CTCs based on size and deformability; however, it may underrepresent smaller or EMT-transitioned CTC-subpopulations. Both of these standard methods have limitations of low capture rate, loss of mesenchymal phenotype and loss of heterotypic clusters. These constraints contribute to the underestimation of biologically relevant CTC subpopulations and limit translational interpretation. Moreover, both have time-consuming analysis, FDA clearance for limited cancers, and are associated with high operational costs^17^. To address these unmet analytical and clinical needs, our group previously developed a FACS-based CTCs detection/isolation strategy, where there is immuno-magnetic depletion of CD45-positive peripheral blood mononuclear cells (PBMCs), which were further positively selected by FACS-based CTC makers (epithelial -EpCAM, Epidermal Growth Factor Receptor (EGFR), CK, as well as mesenchymal markers- (Vimentin) VIM), enabling the detection and isolation of diverse CTC populations^18^. EGFR was included as a tumour-associated marker frequently overexpressed in Oral Squamous Cell Carcinoma (OSCC), while epithelial phenotype classification was primarily based on canonical epithelial markers such as EpCAM and cytokeratins. While this method demonstrated feasibility, sensitivity, and broad applicability, it was primarily optimized for CTC detection and isolation rather than precise enumeration and the platelet-CTC clusters were not systematically resolved.

In the current study, we refined and analytically validated our previous FACS-based method by focusing on the phenotypic heterogeneity (Epithelial and mesenchymal CTCs) and the CTC-platelets heterotypic clusters, which represent the most frequent CTC-associated cell type in circulation^8,11^.

Here, we have designed a specific gating strategy to exclude the events of platelets while selecting the heterotypic clusters of CTC and platelets of both phenotypes, epithelial as well as mesenchymal.

We further demonstrate the clinical applicability of the optimized method by enumerating CTCs in OSCC patients and different fractions of CTCs (Epithelial single CTCs, Mesenchymal single CTC, along with CTC-platelets heterotypic clusters), where validated CTC isolation strategies are lacking.

## Materials and methods

### Healthy controls and patients

10 healthy volunteers without any history of chronic disease, including malignancy, chronic inflammatory conditions, or oral pathological lesions, were recruited for spike-in experiments. 5 mL whole blood in an EDTA-vial (Becton Dickinson, #367856) was collected at three different time points from the same volunteer to check the reproducibility of spike-in experiments and processed within 2 h of collection. Biopsy-proven OSCC cases (*n* = 33) planned for therapy with curative intent (surgery and/or radiotherapy) were recruited from the Department of Radiotherapy and the Department of Otolaryngology, PGIMER, Chandigarh. 5–10 mL peripheral blood sample in an EDTA-vial (Becton Dickinson, #367525) was collected for CTC-count. Informed written consent was obtained from all participants, and the study was approved by the “*Institutional Ethics Committee*” of Postgraduate Institute of Medical Education and Research, Chandigarh vide letter no #IEC-07/2022–2510. The study was also conducted in accordance with the 1964 Declaration of Helsinki and its later amendments. All experiments were performed in accordance with the relevant guidelines and regulations.

### Cell culture

The human OSCC cell line Cal27 (CRL-2095™, ATCC^®^) was used in spike-in experiments. Cells were maintained at 37 °C, 5% CO_2_ in Dulbecco’s Modified Eagle Medium (DMEM) (Lonza, #12–604 F) supplemented with 10% Foetal Bovine Serum (FBS) (Gibco, #10270106) and Antibiotic-Antimycotic (Gibco, #15240062).

### Cell line spike-in experiments

**Fig. 1:**
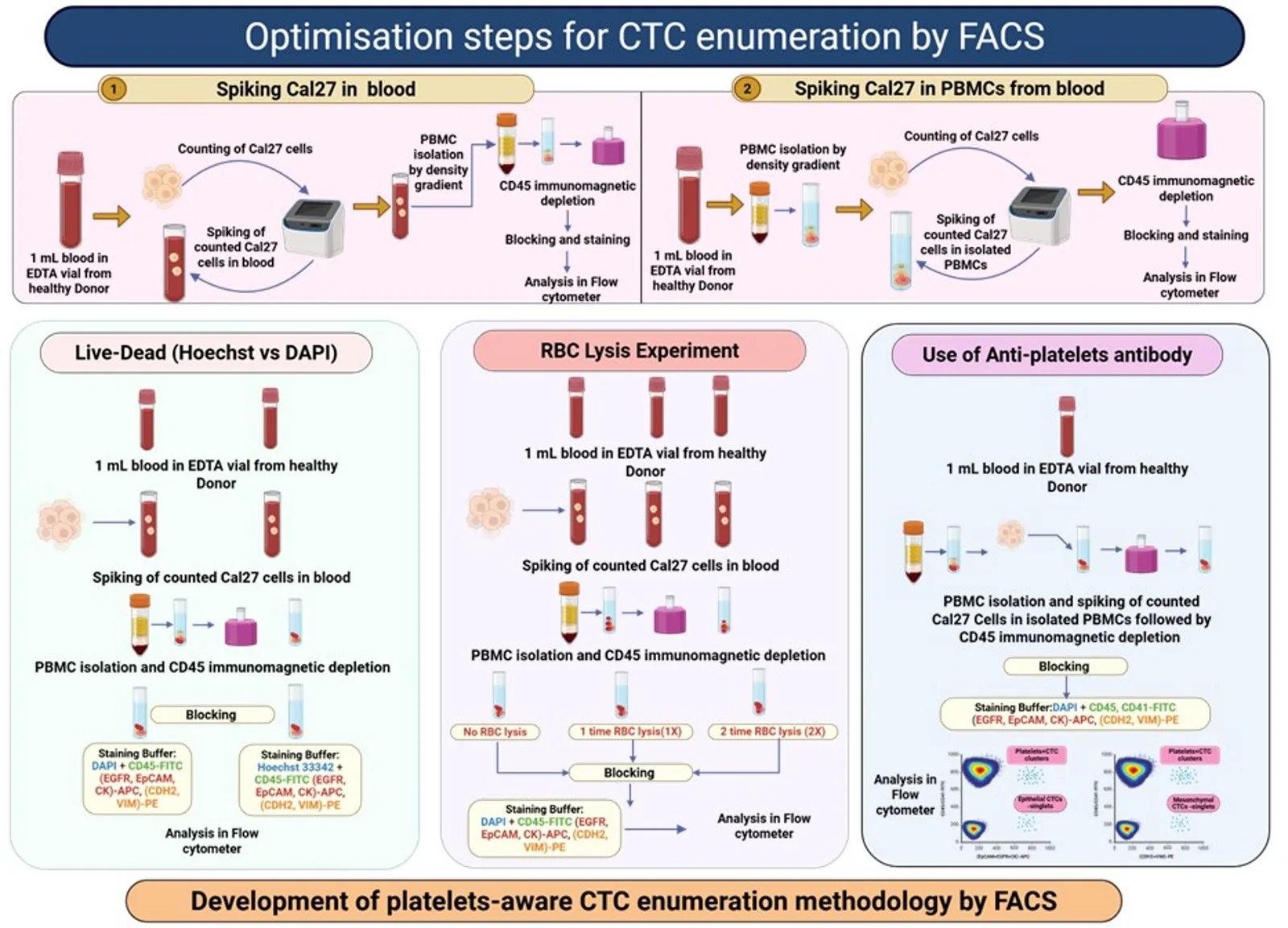
A graphical representation of the spike-in strategy. (Created with BioRender.com).

Cal27 cells were harvested using trypsin (0.25%) (Gibco, 25200072), stained with trypan blue (1:1) and counted in triplicate using the Countess3 Automated Cell Counter (Invitrogen™, #A49865). Serial dilutions were prepared, and 1, 10, or 100 Cal27 cells were spiked in the blood or in the PBMC fraction obtained from 1mL of healthy donor blood, depending upon the experimental requirements (Fig. 1).

### Flow-cytometry

The blood was diluted (1:1) with PBS-2mM EDTA. Cal27 cells were spiked in the blood or PBMCs, as per the experiment requirement. The diluted blood was then carefully layered over 1 mL of HiSep™ LSM 1077 (HiMedia, catalog no. LS001) in sterile 15 mL conical centrifuge tubes, ensuring minimal disturbance of the interface between the blood and the density gradient medium. Following centrifugation, a distinct PBMC layer, located at the plasma–density gradient interface, was carefully identified and gently aspirated using a sterile pipette to avoid contamination with plasma or red blood cells. The collected PBMC fraction was then transferred to fresh tubes, washed and resuspended in the recommended media (2%FBS in PBS-EDTA). PBMCs were then processed for CD45 magnetic depletion using the EasySep™Human CD45 Depletion Kit II (Stemcell Technologies, #17898). Blocking was performed after CD45 depletion to reduce non-specific signal by blocking Fc receptors with a blocking buffer (10% human-serum, 2% BSA, and PBS- 2mM EDTA) for 45 min at 4 °C. After blocking, cells were stained for 1 h at 4 °C (Details of the staining buffer are given in Supplementary Method 1). Cells were resuspended in PBS–EDTA, and analyzed on flow-cytometer/FACS (Navios, Beckman Coulter; BD FACSMelody™). Data were analyzed using Kaluza version 2.1(Beckman Coulter).

Post-staining, cells were resuspended in 300µL PBS–EDTA. After the acquisition, the remaining volume was measured, and volume correction was done for accurate enumeration of CTCs/spiked cells.

### Refining the fluorescence markers

To enable the detection of heterogeneous (CTCs), cell-surface markers were tagged with fluorochromes selected for expression level and signal brightness. Epithelial markers (EpCAM, EGFR, CK) and mesenchymal markers VIM, N-cadherin (CDH2) were labelled with bright fluorochromes APC and PE, respectively. The hematopoietic marker CD45 (for PBMC-exclusion) and CD41 (strategic exclusion of platelets) were labelled with FITC. Hoechst-33,342/DAPI were used to exclude dead cells^19^.

### Molecular confirmation of sorted CTCs

Bulk pools of sorted cells were immediately subjected to Whole Transcriptome Amplification (WTA) using the REPLI-g WTA Single Cell Kit (Qiagen, #50063). The amplified product was diluted (1:100) and analyzed by qPCR for EpCAM, EGFR, CK-19, VIM, CDH2, CD45, and ACTB, in 25µL reaction using 10µL diluted cDNA (details of primers and amplification temperature given in the Supplementary Table 1).

### Evaluation of analytical parameters

We determined the Limit of Detection (LoD) and linearity by spike-in experiments with 1, 10, and 100 Cal27 cells/mL. Specificity and LoD were determined by molecular validation of sorted events by WTA-qPCR. For determining the Limit of Quantification (LoQ), 2, 4 and 8 Cal27 cells were spiked in the PBMCs of 1mL of blood, and the events detected were recorded and compared with the actual spike-in cell count. To determine the Limit of Blank (LoB), 10 mL of blood was processed from healthy donor controls without any spike-in of cells^20^.

Receiver operating characteristic (ROC) analysis was done to set a clinical cutoff for OSCC CTC-positive patients at risk. Youden Index (J) was calculated by the formula- Sensitivity+Specificity-1. J_max_ was used to define the optimal threshold.

### Statistical analysis

Statistical analyses were carried out using GraphPad Prism (version 10) and R software (version 4.4.2). Two-group comparisons used the Mann–Whitney test, while multiple groups were analyzed by the Kruskal–Wallis test. Linearity was assessed using Spearman’s correlation. Data are presented as median (IQR) or mean ± SD, as appropriate. ROC analysis was used to evaluate diagnostic performance, with *p* < 0.05 considered statistically significant.

## Results

### Optimization of gating strategy

**Fig. 2:**
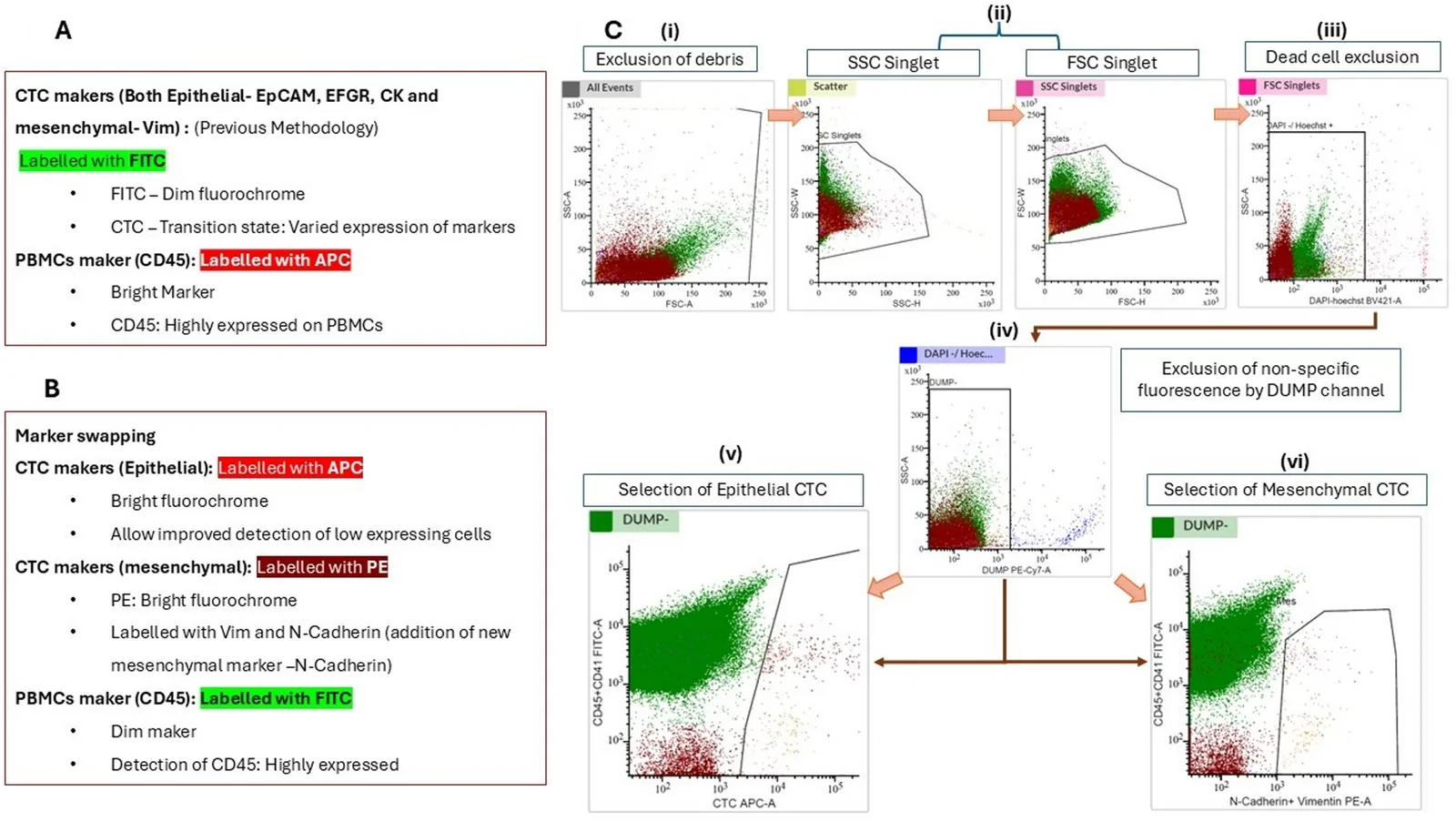
Optimization of FACS-based detection of heterogeneous and rare CTCs (**A**) Original method with – FITC-labelled Epithelial markers (EpCAM, EGFR, CK) as well as mesenchymal marker (VIM) and CD45 tagged with APC (**B**) Optimized gating strategy with bright fluorochromes (APC and PE) separately for Epithelial markers (APC) and mesenchymal markers (PE) while CD45 and CD41 (platelets) tagged with FITC (**C**) Final gating strategy include (i) Forward Scatter (FSC)/Side Scatter (SSC) gating to remove debris, (ii) doublet discrimination gates by plotting SSC-Height (SSC-H) against SSC-Width (SSC-W) along with FSC-Height (FSC-H) vs. FSC-Width (FSC-W) to select singlet population, (iii) exclusion of dead cells using DAPI negative gate, (iv) DUMP gate on PE-Cy7 negative population to remove any non-specific falsefalsefalse fluorescence. (v and vi) Following these preprocessing steps, dual-fluorochrome gating logic was applied to resolve distinct CTC populations. (v) In this gate, APC fluorescence represented epithelial tumour-associated markers (EpCAM, EGFR, and CK), plotted against FITC fluorescence, which contained both CD45 and CD41 signals. APC-positive/FITC-negative events were classified as epithelial single (E-single) CTCs, whereas APC-positive/FITC-positive events were specifically retained and classified as platelet-associated heterotypic CTC clusters. (vi) Similarly, in this gate, PE fluorescence represented mesenchymal markers Vimentin (VIM) and N-Cadherin (CDH2) plotted against FITC channel (CD45 and CD41). PE-positive/FITC-negative events were classified as mesenchymal single (M-Single) CTCs, respectively. Also, PE-positive/FITC-positive events were classified as platelet-associated heterotypic CTC clusters where CTCs are of mesenchymal phenotype. Leukocyte-derived events were identified as FITC+ populations lacking any tumour-marker positivity.

In our previously reported methodology, the cell-surface markers of CTCs (EpCAM, EGFR, CK, and VIM) were tagged with FITC, and for PBMCs, CD45-APC was used (Fig. 2A)^18^. As the cell-surface makers of CTCs present a broad spectrum of gene expression, combining with their extreme rarity, necessitates the use of high-brightness fluorochromes to improve sensitivity^21–23^. Accordingly, we redesigned the antibody panel by conjugating individual epithelial markers (EpCAM, EGFR, CK) to APC and individual mesenchymal markers (VIM, CDH2) to PE, while tagging CD45 (Hematopoietic cell marker) and CD41 (platelet marker) with FITC (Fig. 2B). This configuration maximized signal separation between tumour cells and background leukocytes or platelets. The complete gating strategy for the new antibody panel for sorting CTCs with epithelial as well as mesenchymal phenotypes, along with their respective clusters with platelets, is presented in Fig. 2C.

### Optimization of CTC-enumeration

#### Effect of live-cell selection

**Fig. 3:**
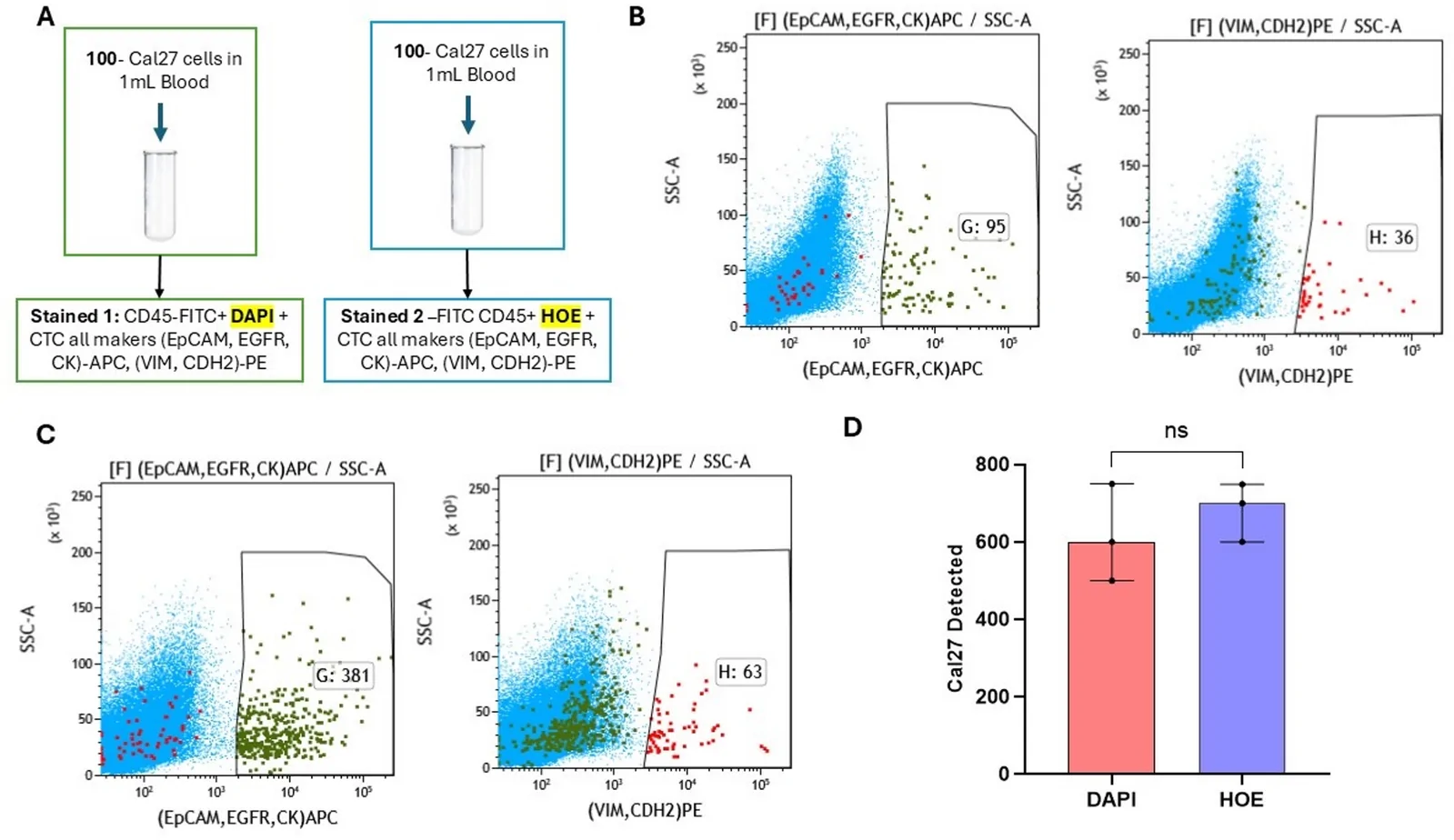
Comparison of dead-cell exclusion and live-cell selection strategies for CTC detection (**A**) Experimental design illustrating spike-in of 100 Cal27 cells in one mL blood of healthy donor, and use of two different staining approaches: Stained 1 includes the use of DAPI for exclusion of dead cells while stained 2 having HOE dye for selection live cells along with other antibodies (CD45-FITC, EpCAM, EGFR and CK labelled with APC along with VIM and CDH2 labelled with PE); (**B**) representative flow-cytometry dot plots for events detected in stained 1- by exclusion the dead cells (DAPI positive exclusion). The first gate is identifies APC-positive (EpCAM, EGFR, CK) epithelial events (green) and second gate is representing PE-Positive (Vim and CDH2) mesenchymal events (red); (**C**) representative dot plots for events detected in stained 2- by inclusion of the live cells (HOE positive inclusion); The first gate is identifies APC-positive (EpCAM, EGFR, CK) epithelial events (green) and second gate is representing PE-Positive (VIM and CDH2) mesenchymal events (red); (**D**) Quantitative comparison of tumor cell related events detected following DAPI-based dead-cell exclusion versus HOE–based live-cell inclusion. Data are presented from three independent experiments per group and were analyzed using the Mann–Whitney U test.

Firstly, to remove false-positive events, we evaluated live-cell selection using Hoechst-33,342 (HOE) in place of dead-cell exclusion with DAPI. Following spike-in of 100 Cal27 cells/mL of healthy donor blood, samples were analyzed using either DAPI (Stained 1) or HOE (Stained 2) separately, along with the other antibodies (Fig. 3A). It was found that there is no difference in the count of detected Cal27 when using HOE (Median 700 cells/mL, IQR 600–749) as compared to DAPI (Median 600 cells/mL, IQR 500–750) (*p* = 0.800) (Fig. 3B-D). The total number of events detected exceeded the number of spiked cells for both DAPI and HOE, suggesting background interference.

#### Contribution of residual RBCs to inflated CTC-counts in flow-cytometry

**Fig. 4:**
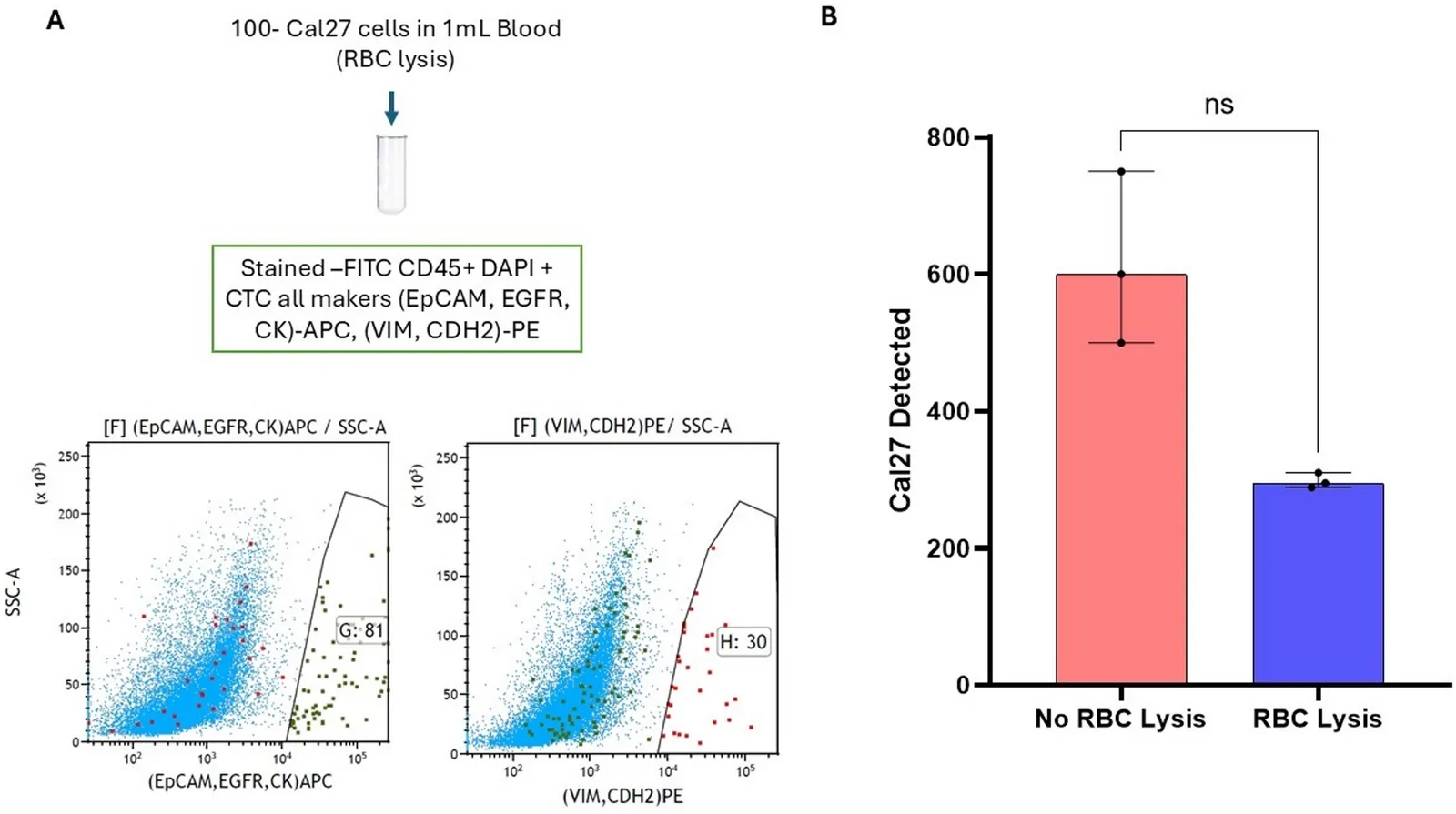
Evaluation of the effect of RBC lysis on the events detection by FACS (**A**) Experimental workflow illustrating spike-in of 100 Cal27 cells in one mL blood, followed by RBC lysis after CD45 immuno-magnetic depletion; In the staining buffer, CD45-FITC, DAPI, and CTC-specific antibodies, including EpCAM, EGFR, and CK conjugated to APC, along with VIM and CDH2 conjugated to PE were used. Representative flow-cytometry dot plots of events detected after RBC lysis: APC-positive epithelial marker-expressing events (EpCAM, EGFR, and CK; left panel- green dots) and PE-positive mesenchymal marker-expressing events (VIM and CDH2; right panel-red dots) are shown as gated populations (green-epithelial events, red-mesenchymal events). Numbers indicate the total events detected within each gate; (**B**) Quantitative comparison of tumour cell–related events detected by flow-cytometry in samples processed with and without RBC lysis, demonstrating a decrease in the number of tumour cell-related events detected by flow-cytometry following RBC lysis. Data are presented from three independent experiments per group and were analyzed using the Mann–Whitney U test.

To improve further, we considered that RBCs, being heme-containing cells, generate noise fluorescence in flow-cytometry and could be one of the reasons for false signals. Despite density-gradient separation, a fraction of low-density RBCs can co-segregate with the PBMC layer. For this, RBC lysis was performed after isolating PBMCs (Supplementary Method 2), followed by CD45 magnetic-depletion, blocking, and staining (DAPI as a dead exclusion dye) (Fig. 4A). The count detected was reduced when RBC lysis was performed (Median 295 Cal27/mL, IQR 289–310) as compared to without lysis (Median 600 Cal27/mL, IQR 500–750) (Fig. 4B), yet counts remained higher than the actual spike-in, suggesting additional confounding factors.

#### Platelets as a major contributor in flow-cytometric CTC-enumeration

Based on the above results, it was evident that other types of cells were influencing the CTC-related count. After RBC-lysis and CD45-depletion, there is only one element left, i.e., Platelets, which might be a cause of high CTC events. This is likely because platelets reside immediately above the PBMC-layer within the platelet-rich plasma during density gradient separation and are inadvertently co-aspirated during PBMC collection (Supplementary Fig. 1). Given their strong propensity to aggregate with tumour cells and generate spurious signals, platelets were hypothesized to be a major source of CTC overestimation. We performed a set of experiments to exclude platelets using a platelet-specific antibody (CD41-FITC) in FACS after RBC-lysis. Platelet exclusion using CD41 gating after one (1X) and two (2X) time RBC lysis resulted in markedly reduced recovery, likely due to cumulative cell loss during lysis and subsequent wash steps (Supplementary Fig. 2A, 2B). The RBC-lysis step includes two PBS washes, which may cause loss of spiked cancer cells and result in low event counts. To check this, we used only CD41 platelets removal without RBC-lysis, which substantially improved recovery (average count ~ 351 when 1000 Cal27 were spiked/mL of blood) (Supplementary Fig. 2C, 2D and Supplementary Fig. 3). The detection efficiency remained low, which is around 30%, potentially due to complement-mediated tumour cell loss in whole blood. To address this, Cal27 cells were spiked directly in the PBMC-layer isolated from 1mL of blood, followed by CD45-depletion, blocking, staining with the staining cocktail ( CD45-FITC + CD41-FITC + DAPI + EpCAM-APC, EGFR-APC, CK-APC, VIM-PE, CDH2-PE) and then analyzed in the flow-cytometer, which leads to a significantly improved detection efficiency (735 events out of 1000 Cal27 spiked, Supplementary Fig. 4).

### Analytical performance of the optimized assay

In the next step, we established linearity, Limit of Detection (LoD), Limit of Quantification (LoQ) and Limit of Blank (LoB) of this method. For this, we spiked 100, 10, and 1 Cal27 in the PBMC-layer isolated from 1mL blood and proceeded for flow-cytometric analysis according to the optimized protocol and Cal27 events were enumerated for subsequent analysis.

#### Linearity

**Fig. 5:**
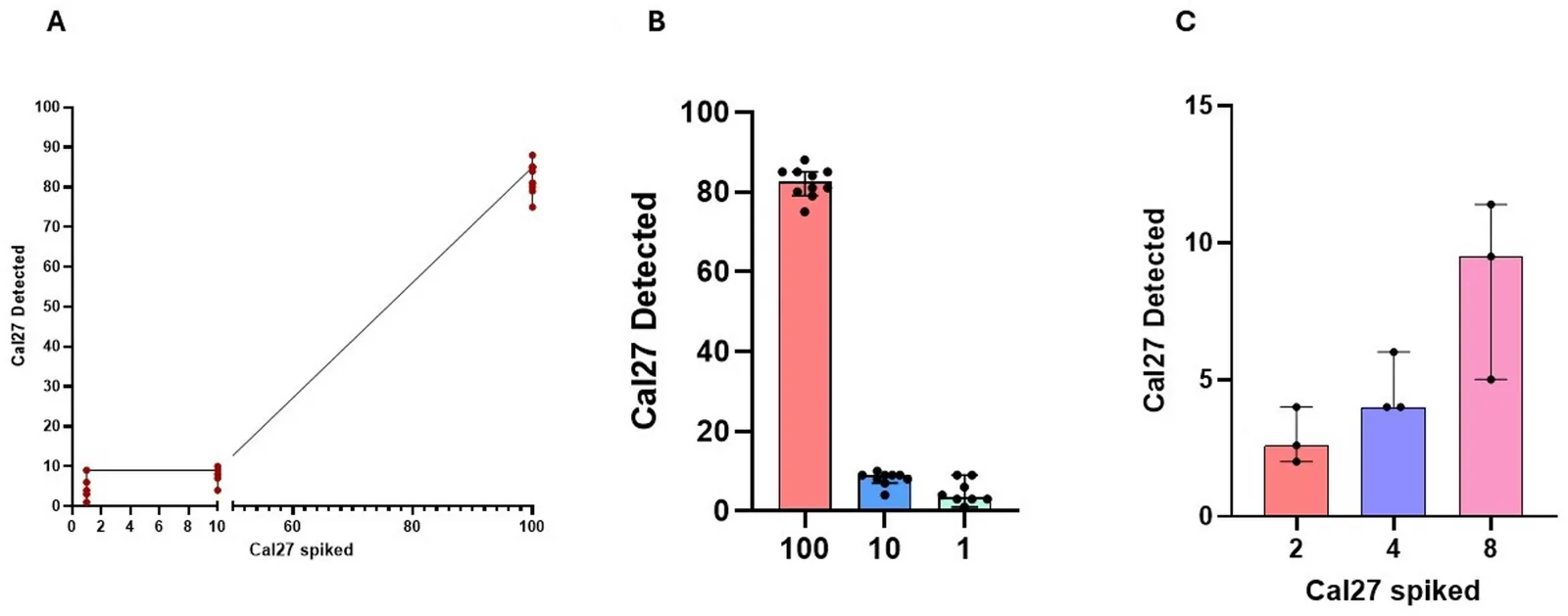
Assessment of analytical parameters of the optimized method (**A**) linearity assessment by spike-in increasing numbers of Cal27 cells (1, 10, and 100 cells) into the PBMC fraction obtained from 1 mL of blood and comparing the number of cells detected by flow-cytometry. A strong linear correlation was observed by Spearman’s correlation analysis. For 100 cells, the experiment was performed in 10 replicates, for 10 cells ( 9 replicates) and for 1 cell (8 replicates); (**B**) numbers of Cal27 detected following spike-in of 100, 10 and 1 Cal27 cells in the PBMCs fraction of 1mL blood. This was used to calculate the detection efficiency and LoD. Bar graphs represent the mean number of Cal27 cells detected, while individual dots correspond to independent replicates. The assay consistently detected Cal27 cells up to single-cell spike-in in one mL, demonstrating high analytical sensitivity. For 100 cells, the experiment was performed in 10 replicates, for 10 cells ( 9 replicates) and for 1 cell (8 replicates). The data were analysed by the Kruskal–Wallis test; (**C**) assessment of LoQ using low-level spike-in experiments with 2, 4, and 8 Cal27 cells. Bars represent the median number of detected cells, and error bars indicate standard deviation. Data are presented from three independent experiments per group and were analyzed using the Kruskal–Wallis test.

To check the linearity, the number of Cal27 cells detected in flow-cytometric analysis was correlated with the number of spiked cells. A strong linear correlation was observed with a Spearman’s correlation coefficient of 0.8738, with 95% CI of 0.7338–0.9426 (*p* < 0.0001) (Fig. 5A).

#### Limit of detection (LoD)

100, 10 and 1 Cal27 cells were spiked in the PBMC fraction of 1mL blood and sorted by FACS using the optimized strategy. Immediately after sorting, WTA was performed to amplify the cDNA directly from sorted cells, and qPCR was performed for the CTC-markers (EpCAM, EGFR, Cytokeratin-19 (CK-19), vimentin (VIM), and N-cadherin (CDH2)) with CD45 to qualitatively confirm the purity of detected events and PBMC removal. This has been repeated in 3 independent experiments. All the sorted events showed specific positivity for CTC-markers and were negative for CD45 (Supplementary Tables 2 and Supplementary Fig. 5). It was observed that even a single spiked cell was detectable and isolated by our protocol, demonstrating a LoD of 1 cell/mL.

#### Detection efficiency

Detection efficiency was calculated by comparing detected versus spiked cell numbers. Mean detection efficiency was 82.30 ± 3.80% for 100 cells and 81.11 ± 17.64% for 10 cells. Further, when 1 Cal27 cell was spiked, detection occurred in all experiments, but the number of detected cells was more than one (average count 4.7 ± 2.964) (Fig. 5B and supplemental Fig. 6). This might be because of an unavoidable error in counting before spike-in of Cal27, and further manual pipetting error may pick more than one cell or may be due to the detection of cell fragments as an event during acquisition by flow-cytometry.

#### Limit of quantification (LoQ)

Although using the method, even one cell per mL of blood can be detected, the LoQ was evaluated by spiking 2, 4, and 8 Cal27 cells in the PBMCs isolated from 1 mL of blood. The median counts detected were 2.6 cells/mL with 0.3171–5.416 CI (for 2 cells spiked), 4 cells/mL with 1.798–7.535 CI (for 4 cells spiked) and 9.5 cells/mL (95% CI: 0.47–16.8) (for 8 cells spiked) (Fig. 5C and Supplementary Fig. 7). These results indicated increased variability at low cell numbers; therefore, quantification with acceptable precision and reproducibility was achieved at ≥ 10 cells/mL, establishing the LoQ.

#### Limit of Blank (LoB)

**Fig. 6:**
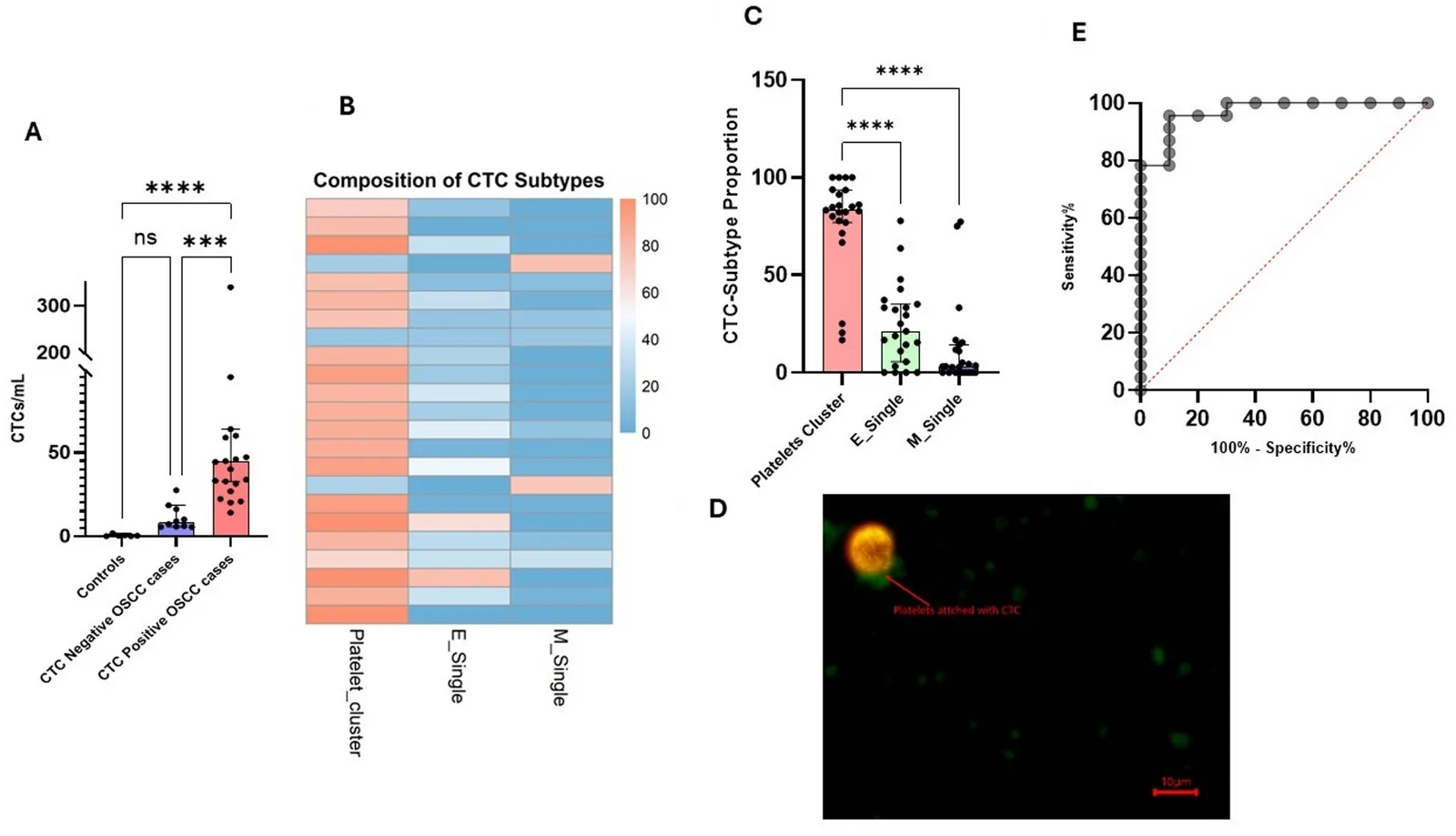
CTC enumeration and CTC-subtype compositions in OSCC patients (**A**) CTC count detected by the developed method compared between control and OSCC patients. CTC-positive patients (*n* = 23) have significantly higher CTC-related counts as compared to CTC-negative patients (*n* = 10) as well as the healthy controls (*n* = 6). Quantitative comparison has been done by the Kruskal-Wallis test, presented with median and 95% CI; (**B**) Heatmap showing composition of different subtypes of CTCs in OSCC patients. Higher composition of platelet-associated CTCs was found as compared to the epithelial single (E-Single) CTCs, and mesenchymal single (M-Single) CTCs across OSCC patients; (**C**) Kruskal Wallis test for quantitative comparison of CTC subtype proportions revealed significant high proportion of CTC-platelet clusters (Median- 83.33%; 95% CI 67.15–88.15) as compared to E-Single CTCs (Median- 21.21%; 95% CI 15.55–33.47) (*p* < 0.0001), as well as M-Single CTCs (Median- 3.226%; 95% CI 2.765–21.55) (*p* < 0.0001); (**D**) Immunofluorescence showing clusters of platelets (Green, CD41-FITC) attached on the surface of CTC (Red, EpCAM, EGFR, CytoKeratin tagged with APC and VIM, CDH2 tagged with PE) at resolution 40X; (**E**) ROC analysis comparing CTC-counts between the CTC-positive and CTC-negative patients, yielding an AUC of 0.9696 and identifying > 19.40 FACS-detected CTC-related events/mL of blood as the optimal cutoff point.

To establish the LoB, 10 mL of blood from healthy donors (*n* = 6) was processed using the optimized method. The detected CTC-like events range from 0.2 to 2 events/mL, with a median of 0.4 events/mL (0.2–1.025 95% CI). Importantly, none of the sorted events from healthy donors showed positivity for CTC-markers by qPCR (Supplementary Tables 3 and Fig. 6A). To set the LoB, we used the following formula^20^.

$${\mathrm{LoB}} = {\mathrm{mean}}_{{{\mathrm{blank}}}} + {\mathrm{1}}.{\mathrm{645}}\left( {{\mathrm{SD}}_{{{\mathrm{blank}}}} } \right)$$

A mean of 0.65 events/mL (SD:0.6863), the LoB was calculated to be 1.779 events/mL. LoB indicates a threshold for the background noise and the highest CTC-related event counts, which may be detected from the blood of a healthy donor.

### Application to patient samples

The modified strategy enabled consistent detection of CTCs in OSCC patients ranging from 5.6 to 340.5 cells per mL of blood. Clinical details of all patients are given in Supplementary Table 4. The CTCs were sorted and proceeded to WTA-qPCR, for confirmation of CTCs markers and CD45. The patients showing positivity for at least one CTC marker were classified as CTC-positive. Out of 33 OSCC cases, 23 were classified as CTC-positive (70%) while 10 were CTC-negative (30%). CTC-positive patients exhibited significantly higher CTC-related event counts as compared to both CTC-negative patients and healthy controls (***p = 0.0005*** and ***0.0002***). Also, no significant difference in counts of CTC-related events was observed between CTC-negative OSCC cases and healthy controls (*p* = 0.6876), Fig. 6A. In CTC-positive patients, the number of positive events detected ranges from 14.20 to 340.5 per mL (median 45.00 and 95% CI 37.84–97.76). In CTC-negative cases, event ranges from 5.6 to 27.60 per mL (median 8.30 and 95% CI: 6.014–16.51). Whereas healthy donors exhibited low background counts ranging from 0.4 to 2.0 cells/mL (median 0.75 cells/mL; 95% CI: 0.15–2.10), Fig. 6A.

After the total count, we evaluated the proportion of different CTC subpopulations in OSCC patients from the recorded event data of FACS. The percentage of platelet-associated CTC clusters, epithelial single (E-Single) CTCs, and mesenchymal single (M-Single) CTCs relative to total CTC counts for each patient was calculated. The heatmap visualization revealed a heterogeneous CTC-subpopulation/states across patients. However, platelet-associated CTC clusters were the predominant fraction in each patient’s sample. In contrast, E-Single and M-Single CTCs comprised a smaller proportion of the total CTC pool (Fig. 6B). Statistical analysis demonstrated that the proportion of CTC-platelet heterotypic clusters was significantly higher (83.33%) compared to E-single (21.21%, *p* < 0.0001) and M-single CTCs (3.226%, *p* < 0.0001) (Fig. 6C). These findings indicate that platelet-associated CTC clusters represent the predominant circulating tumour cell phenotype, followed by E-Single CTCs, while M-Single CTCs constitute the least abundant population. Platelet attachment to CTCs was further confirmed by immunofluorescence imaging of isolated CTCs, where platelet-CTC clusters were clearly observed (Fig. 6D). Collectively, these results highlighted the biological and analytical utility of the inclusion of platelet-associated CTC clusters with dual FACS gating and simultaneous evaluation of both epithelial and mesenchymal phenotypes to fully capture the CTC heterogeneity. Further, to establish an optimal cutoff for CTC counts to differentiate between CTC-positive and CTC-negative OSCC, we assessed optimal sensitivity and specificity by analyzing an ROC curve. The assay demonstrated good diagnostic performance with an AUC of the ROC of 0.9696. A cutoff of > 19.40 events was determined using the maximum Youden Index (J_max_), with 95.65% sensitivity and 90% specificity, and was selected as the threshold for CTC-positive OSCC patients (Fig. 6E).

## Discussion

Accurate detection and enumeration of CTCs is crucial for the identification of early metastasis, disease monitoring, therapeutic response assessment, and personalized treatment planning^24–26^. The current methods have advanced the field, but have a number of limitations, including the inability to capture EMT-transitioned CTCs with low EpCAM expression, loss of cell viability due to fixatives (for microscopy and enumeration), contamination with leukocytes, which compromises purity and analytical accuracy, and under-recognition of CTC heterotypic clusters^27–31^. Moreover, many of these existing CTC detection platforms lack a well-standardized and clinically validated workflow, and their high operational and instrumentation costs limit their widespread adoption, particularly in Low- and Middle-Income Countries (LMICs).

Our previously developed FACS-based method was able to detect and isolate live and heterogeneous CTCs^18^. In the present study, we optimised our FACS-based approach to accurately enumerate different CTC states -Epithelial CTC, mesenchymal CTC and CTC-platelets clusters. We interrogated and systematically optimized multiple technical factors that contribute to false-positive events and suboptimal recovery, enhancing both detection efficiency and assay robustness for accurate enumeration.

One of the key improvements in our methodology was the reassignment of fluorochromes, overcoming the signal loss due to low antigen density on the cell surface of CTCs^23^. We further addressed false-positive events arising from dead cells and debris by incorporating Hoechst-33342 for live-cell selection, from RBCs by lysing them and from platelets by adding CD41-FITC antibodies, sequentially. The Hoechst-based live-cell discrimination gating did not significantly change the Cal27 detection compared to DAPI-based dead-cell exclusion, confirming that dead cells were not the primary contributors to background events. Instead, these findings suggested the presence of additional blood-derived components associated with high event detection by FACS. RBC-lysis after CD45 immuno-magnetic depletion partially reduced background events, but also led to a substantial loss of spiked tumour cells, which may be because of additional centrifugation and washing steps.

Importantly, our results demonstrated that a significant number of platelets persist even after RBC-lysis and CD45-depletion, which further leads to an increase in the false-positive detection due to their size, abundance, and ability to bind cancer cells. The results highlighted two distinct clusters in the flow-cytometer, one of an APC-only positive cluster (APC⁺/FITC⁻), representing E-Single CTCs (Cal27-singlets) and a second cluster of a double-positive cluster (APC+/FITC+), representing heterotypic aggregates of Cal27 bound to platelets. For mesenchymal CTCs, a similar clustering was observed. This approach allows the precise identification of both epithelial and mesenchymal CTC subpopulations, along with their heterotypic clusters with platelets. It was also evident from our results that the major proportion of CTCs (83.33%) was associated with platelets to form heterotypic clusters in the patients. Hence, including only epithelial and mesenchymal markers will lead to the loss of the major CTC sub-population.

Conventional platforms such as the CellSearch System have reported CTC positivity rates of approximately 25% in Head and Neck Squamous Cell Carcinoma (HNSCC), using a threshold of ≥ 1 CTC per 7.5 mL of blood^32^. Similarly, Parsortix technology, another FDA-approved technology, is biased toward capturing epithelial-like CTCs and has a mean capture rate of 53.5%^33^. In contrast, by incorporating platelet-inclusive dual gating and resolving heterotypic clusters, our optimized workflow achieved a 70% CTC detection rate in OSCC patients at diagnosis. This enhanced positivity rate likely reflects more comprehensive biological capture rather than analytical inflation, as platelet-associated CTCs represent a dominant and clinically relevant circulating subpopulation. Previous studies have also reported the presence of CTC-platelet heterotypic clusters and have demonstrated that these platelet-associated CTCs have enhanced metastatic potential^10,11,34–36^. The analytical performance evaluation demonstrated that the assay is highly robust and sensitive. It showed strong linearity with a high correlation between expected and observed counts (*r* = 0.8738) across different cell numbers spiked. Along with this, LoD was determined to be 1 cell/mL, confirming the ability of the method to detect extremely rare events. The LoQ was found to be 10 cells/mL. At extremely low spike-in concentrations (1–10 cells), stochastic variability associated with serial dilution and manual pipetting may affect absolute quantitative accuracy. Therefore, the LoD should be interpreted as demonstrating rare-event detectability rather than precise quantification at single-cell resolution. Additionally, LoB is essential for distinguishing true biological events from background noise. LoB was established at 1.779 cells/mL based on healthy donor samples, with negative molecular confirmation of CTC identity by WTA, which provided a clear, stringent threshold for distinguishing true biological events from background noise.

When the optimized method was applied to clinical samples from OSCC patients, CTCs were detected in 70% of patients at diagnosis, with counts (14.20–340.5 CTCs/mL) significantly higher than both CTC-negative OSCC patients and healthy controls. Molecular confirmation of CTC identity further strengthened these findings. Importantly, ROC curve analysis yielded an AUC of 0.9696, allowing us to define an optimal cutoff of > 19.40 CTCs/mL for CTC positivity with 95.65% sensitivity and 90.00% specificity. This clear separation between CTC-positive and CTC-negative cases highlights the potential utility of this assay for clinical stratification. The ROC-derived threshold was generated using the same exploratory cohort used for CTC classification, which may introduce overfitting. Therefore, the proposed cutoff requires validation in larger independent cohorts. However, future studies incorporating normalization strategies based on PBMC recovery, total nucleated cell counts, or volumetric controls will be important to enhance inter-sample comparability and analytical precision.

By resolving circulating tumour cell heterogeneity within a pathology-oriented and quantitatively validated framework, this study advances both biological understanding and translational applicability in OSCC. Unlike marker-restricted or size-based platforms, this method captures viable CTCs across epithelial and mesenchymal phenotypes, including their platelet-associated clusters, thereby providing a more comprehensive representation of the circulating tumour cell pool.

Although platelet-associated CTCs represented the major fraction in our analysis, it is important to consider that pre-analytical and analytical factors may influence their relative abundance. Platelets are known to readily associate with tumor cells, and such interactions may be preserved or potentially enhanced during sample processing. However, the consistent detection across patient samples, along with immunofluorescence-based confirmation and prior biological evidence, supports the interpretation that these clusters represent a biologically relevant circulating phenotype rather than solely a technical artifact.

Certain limitations of our study are also acknowledged. The current study focuses on baseline enumeration, and longitudinal follow-up studies are needed to correlate distinct CTC states with recurrence, therapeutic response, and survival outcomes. Additionally, while platelet-associated clusters were prioritized due to their predominance and biological significance, future studies should expand investigation to other heterotypic CTC interactions within the tumour–immune microenvironment. Another limitation is that we could not analyse the same sample in parallel by our strategy, vis-à-vis by CellSearch or Parsortix, as these were unavailable. In conclusion, this study presents a highly sensitive and specific FACS-based CTC detection, enumeration and isolation method that addresses the key challenges of CTC rarity, heterogeneity, false-positive artefacts and CTC-platelets heterotypic cluster. Analytical and molecular validation demonstrates high recovery efficiency, low limits of detection and quantification, and reliable enumeration across clinically relevant CTC concentrations. Our method detects CTCs with varying degrees of epithelial and mesenchymal phenotypes along with their heterotypic clusters with platelets, which remained unaddressed by the majorly available platforms. The method is suitable for cancer prognostication and disease monitoring and can be expanded to other cancers by changing the markers.

## Supplementary Information

Below is the link to the electronic supplementary material.

Supplementary Material 1

## Data Availability

All data generated or analysed during this study are included in this published article [and its supplementary information files]. Moreover, the raw data generated and/or analysed during the current study available from the corresponding author on reasonable request also.
