# Supplementary material for: Identification of epithelial, mesenchymal, and platelet-associated circulating tumour cells with translational implications in oral squamous cell carcinoma
Source: Sci Rep. 2026 Jun 10;16:26281. doi: 10.1038/s41598-026-57427-z (PMC13493767; doi:10.1038/s41598-026-57427-z)
Supplement: Supplementary file 1 [file 41598_2026_57427_MOESM1_ESM.docx]

**Supplementary Methods:**

**Supplementary Method 1:**

*Antibody cocktail:*

i. Cytokeratin Pan Monoclonal Antibody (C-11), APC, ThermoFisher Scientific, Cat #MA1-10325
ii. APC anti-human EGFR Antibody (AY13): 352906 -Biolegend
iii. CD326 (EpCAM) Monoclonal Antibody (9C4), APC, ThermoFisher Scientific, Cat No.- 324208
iv. CD325 (N-Cadherin) Monoclonal Antibody (8C11), PE, ThermoFisher Scientific, Cat #12-3259-42
v. CD45 Monoclonal Antibody (HI30), FITC, eBioscience™ Cat # 11-0459-42
vi. CD41a Monoclonal Antibody (HIP8), FITC, eBioscience™ Cat # 11-0419-42
vii. Vimentin Monoclonal Antibody (VI-RE/1), PE, ThermoFisher Scientific, Cat # MA1-19656

4′,6-Diamidino-2-phenylindole (DAPI) (#D3571, Invitrogen™).

**Supplementary Method 2:**

**RBC lysis:** RBC lysis buffer was prepared (1 Litre) by mixing 8.02 g of Ammonium Chloride (M.W. 53.49), 1 g of Potassium Bicarbonate (M.W. 100.12) and 0.0372 g of Disodium EDTA (M.W. 372.24) in Distilled water. Every time fresh RBC lysis buffer was prepared. 9 mL of RBC lysis buffer was added to 1 mL of cell suspension, incubated at room temperature for 10 minutes on a rocker. After 10 minutes, PBS-2mM EDTA was added to make volume 15 mL. The tube was centrifuged at 400g for 5 minutes at 4 ^°^C, and the supernatant was discarded. The second washing was given similarly, with 10 mL PBS-2mM EDTA. In some experiments, one-time RBC lysis has been done, labelled as -1X RBC lysis, and in other experiments, two cycles of RBC lysis were done, labelled as 2X RBC lysis.

**Supplementary Figures:**

**Supplementary Figure 1:**

*Presence of platelets in the PBMC layer*

**Supplementary Figure 1:** **(A)** PBMCs isolated from 1mL blood of a healthy subject were treated with RBC lysis buffer, followed by staining with DAPI and CD45-FITC antibody, and an unstained control was taken; **(B & C)** Flow-cytometry representative dot-plot of unstained control tube **(B)** and of stained tube **(C),** the first gate represents the total population (FSC-A vs SSC-A), followed by CD41-FITC positive population (orange) showing the presence of contaminating platelets even after RBC lysis of PBMCs in the stained tube.

**Supplementary Figure 2**

*Synergistic effect of RBC lysis and platelet removal*

**Supplementary Figure 2:** Effects of 1X RBC lysis (1 cycle of RBC lysis), 2X RBC lysis (2 cycle of RBC lysis), along with platelets removal in flow-cytometer gating on count of spiked Cal27 detected; **(A)** Spiking of 1000 Cal 27 cells in 1 mL of blood- RBC lysis performed one time (1X RBC lysis) and two times (2X RBC lysis) after CD45 magnetic depletion along with platelet exclusion by adding CD41-FITC in the staining buffer; **(B),** 1000 Cal27 cells were spiked in 1ml of blood, no RBC lysis was done, but the staining buffer includes CD41-FITC antibody along with the others; **(C),** Representative flow-cytometry dot plots illustrating the gating strategy for detection of 1000 spiked Cal27 cells in 1 mL blood following 1X RBC lysis and 2X RBC lysis. The given first plot identifies Cal27 cells singlet (only APC⁺) and its cluster with platelet (APC⁺ and FITC⁺) detected in the APC channel (pink). In contrast, singlet platelets and CD45⁺ PBMCs were excluded as FITC⁺ only signal (orange and blue). In the second plot, these events were subsequently analysed for mesenchymal marker expression, where the H gate represents VIM⁺/CDH2⁺ Cal27 cells detected in the PE channel after platelet exclusion gating. The numbers within the gates indicate the count of detected events under each RBC lysis condition; **(D)** Similar representative flow-cytometry dot plots showing the detection of **1000 spiked Cal27 cells in 1 mL blood without RBC lysis**.

**Supplementary Figure 3**

*Synergistic effect of RBC lysis and platelet removal (CD41 Removal)*

|  |  |  |  |
| --- | --- | --- | --- |
| 1X RBC Lysis +CD41 Removal | 74 | 51 |  |
| 2X RBC Lysis CD41 Removal | 21 |  |  |
| Only CD41 removal +No RBC Lysis | 426 | 304 | 324 |

**Supplementary Figure 3:** Comparison of the count of Cal27 cells detected (in X axis) in the flow-cytometer, using cell spike-in experiments with and without RBC lysis, followed by platelets exclusion gating. 1000 Cal27 cells were spiked in 1mL of blood. Each bar shows an individual experiment.

**Supplementary Figure 4**

*Cal27 cells detected after spiking directly in PBMCs*

**Supplementary Figure 4:** (**A)** Spike-in of 1000 Cal27 cells, directly in the PBMC layer obtained from 1 mL of blood from a healthy donor. RBC lysis was not performed; samples were stained with CD45-FITC and CD41-FITC for exclusion of leukocytes and platelet-associated events, along with DAPI and CTC-specific antibodies (EpCAM, EGFR, CK, tagged with APC and VIM, CDH2 tagged with PE) and recovery of cells was done using the final optimised gating strategy. **(B)** Representative dot plots of flow-cytometer gating. Sequential gating was performed by selecting DAPI-negative events (gate C), followed by exclusion of events of non-specific fluorescence using the DUMP gate (gate G). Subsequently, CD45⁻CD41⁻ events (gate J) were selected to remove hematopoietic and platelet-associated cells. Within the CD45⁻CD41⁻ population, tumor cells expressing epithelial markers (EpCAM/EGFR/CK-APC; gate L) and mesenchymal markers (VIM/CDH2-PE; gate M) were identified. In the final plot of dual gating, APC and FITC were plotted together, where the events expressing only APC (singlet Cal27) and both expressing APC and FITC (Cal27 and platelet clusters) were selected in gate N (red). A total of ~735 Cal27 cells were recovered from the 1000 cells spiked into the blood.

**Supplementary Figure 5**

*Molecular marker reconfirmation of sorted events for determination of LoD (Limit of Detection):*

**Supplementary Figure 5:** Representative qPCR amplification curves of EpCAM, EGFR, CK-19, VIM, CDH2 and CD45 for confirmation of molecular marker positivity in sorted Cal27 when spiked with 100, 10 and 1 cells along with No Cal control.

**Supplementary Figure 6:**

*Determination of detection efficiency:*

**Supplementary Figure 6:** Representative flow-cytometric dot plots showing the detection of Cal27 cells spiked into PBMCs from 1mL of blood at three different concentrations (100, 10, and 1 Cal27/mL). In the dot plots, dual gating approach was used to identify singlet Cal27-related events (APC^+^) and their cluster with platelets (FITC^+^APC^+^).

**Supplementary Figure 7:**

*Determination of LoQ (Limit of Quantification)*

**Supplementary Figure 7:** Representative flow-cytometric dot plots for determination of LoQ. 2, 4, and 8 Cal27 cells spiked into PBMCs from 1mL of blood and detected by dual gating approach by identifying singlet Cal27 related events (APC^+^) and their cluster with platelets (FITC^+^APC^+^).

**Supplementary Tables:**

**Supplementary Table 1:** Sequence of primers and annealing temperature, used for CTC confirmation.

| **Primers** | **Sequence 5’-3’** | Annealing Temperature (degree Celsius) |
| --- | --- | --- |
| EGFR-F | CTCAGCCACCCATATGTACC | 60 |
| EGFR-R | GGTCTCGGGCCATTTTGG |  |
| EpCAM-F | AATGAATGGCTCAAAACTTGG | 55 |
| EpCAM-R | CAGTAGGTTCTCACTCGCTCAG |  |
| CK-19-F | GGTCAGTGTGGAGGTGGATT | 60 |
| CK-19-R | TCAGTAACCTCGGACCTGCT |  |
| VIM-F | TCTACGAGGAGGAGATGCGG | 60 |
| VIM-R | GGTCAAGACGTGCCAGAGA |  |
| CD45-F | CTGACATCATCACCTAGCAG | 60 |
| CD45-R | TGCTGTAGTCAATCCAGTGG |  |
| CDH2-F | CCTCCAGAGTTTACTGCCATGAC | 55 |
| CDH2-R | GTAGGATCTCCGCCACTGATTC |  |
| β-Actin-F | CCCTATAAAACCCAGCGGC | 60 |
| β-Actin-R | AAGGTCTCAAACATGATCTGGGT |  |

**Supplementary Table 2:** Molecular markers positivity by q-PCR in WTA product of sorted cells after spike-in of 100, 10 and 1 Cal 27 cell in PBMCs of 1mL blood. No Cal CTRL: No cells were spiked (Negative control).

|  | **Cell spiked** | | |  |
| --- | --- | --- | --- | --- |
| **Experimental set** | **100** | **10** | **1** | **No Cal CTRL** |
| S17 | EpCAM, EGFR, CK, VIM, CDH2 | EGFR, EpCAM, CK, CDH2 | EGFR, EpCAM, CK | 0 |
| S18-1 | EpCAM, EGFR, CK, VIM, CDH2 | EGFR, EpCAM, VIM, CDH2 | EGFR, EpCAM, CDH2 | 0 |
| S18-2 | EpCAM, EGFR, CK, VIM, CDH2 | EGFR, EpCAM, CDH2 | EGFR, EpCAM, VIM | 0 |
|  | All are CD45 Negative | | | |

**Supplementary Table 3:** CTC-related event count in patients and healthy controls and molecular marker reconfirmation.

| **Sample ID** | **Blood Volume (mL)** | **Total CTC related events** | **CTC related events/mL of blood** | **CTC Marker after WTA and qPCR** |
| --- | --- | --- | --- | --- |
| 1 | 8 | 760 | 95 | CK EPCAM CDH2 |
| 2 | 10 | 445 | 44.5 | CK VIM EPCAM |
| 3 | 10 | 59 | 5.9 | Negative |
| 4 | 8 | 512 | 64 | EPCAM |
| 5 | 10 | 101 | 10.1 | Negative |
| 6 | 10 | 163 | 16.3 | Negative |
| 7 | 10 | 209 | 20.9 | CK VIM EPCAM CDH2 |
| 8 | 10 | 92 | 9.2 | Negative |
| 9 | 10 | 224 | 22.4 | CK CDH2 |
| 10 | 8 | 360 | 45 | CK |
| 11 | 10 | 56 | 5.6 | Negative |
| 12 | 10 | 321 | 40.125 | CK |
| 13 | 10 | 461 | 46.1 | CK |
| 14 | 10 | 472 | 47.2 | CK CDH2 |
| 15 | 8 | 481 | 60.125 | VIM |
| 16 | 10 | 57 | 5.7 | Negative |
| 17 | 8 | 1003 | 125.375 | CK EPCAM CDH2 |
| 18 | 5 | 31 | 6.2 | Negative |
| 19 | 8 | 922 | 115.25 | EPCAM CDH2 |
| 20 | 8 | 1090 | 136.25 | EGFR CK |
| 21 | 8 | 2724 | 340.5 | EPCAM CDH2 |
| 22 | 5 | 295 | 59 | EpCAM CK CDH2 |
| 23 | 5 | 71 | 14.2 | EpCAM CDH2 |
| 24 | 5 | 93 | 18.6 | Negative |
| 25 | 10 | 202 | 20.2 | CDH2 |
| 26 | 10 | 74 | 7.4 | Negative |
| 27 | 10 | 315 | 31.5 | CK |
| 28 | 10 | 339 | 33.9 | CK |
| 29 | 10 | 269 | 26.9 | VIM |
| 30 | 5 | 166 | 33.2 | EpCAM |
| 31 | 5 | 138 | 27.6 | Negative |
| 32 | 5 | 164 | 32.8 | CK |
| 33 | 5 | 525 | 105 | EpCAM CK |
| Control 1 | 10 | 7 | 0.7 | Negative |
| Control 2 | 10 | 4 | 0.4 | Negative |
| Control 3 | 10 | 2 | 2 | Negative |
| Control 4 | 10 | 2 | 0.2 | Negative |
| Control 5 | 10 | 2 | 0.2 | Negative |
| Control 6 | 10 | 4 | 0.4 | Negative |

**Supplementary Table 4:** Details of patients

| **Sample ID** | **Clinical-Stage** | **Age** | **Gender** | **Site** |
| --- | --- | --- | --- | --- |
| 1 | T3N2cM0 | 52 | M | Base of Tongue |
| 2 | T3N2M0 | 57 | M | Oropharynx |
| 3 | T4aN2M0 | 51 | M | Buccal Mucosa |
| 4 | T2N0M0 | 43 | M | Tongue |
| 5 | T2M0M0 | 70 | F | Tongue |
| 6 | T4aN0M0 | 30 | M | Oral Cavity |
| 7 | T2N1M0 | 71 | M | Oropharynx |
| 8 | T4bN2cM0 | 67 | M | Buccal Mucosa |
| 9 | T3N0M0 | 71 | M | Oral Cavity |
| 10 | T4N0M0 | 77 | M | Tongue |
| 11 | T2N1M0 | 60 | M | Base of Tongue |
| 12 | T2N0M0 | 49 | M | Oropharynx |
| 13 | T3N1M0 | 56 | F | Oropharynx |
| 14 | T4aN2cM0 | 63 | M | Oral cavity |
| 15 | T1N1M0 | 40 | M | Oropharynx |
| 16 | T4aN0M0 | 49 | M | Buccal Mucosa |
| 17 | T4N3M0 | 52 | M | Oropharynx |
| 18 | T4bN0M0 | 49 | M | Oral cavity |
| 19 | T1N0M0 | 52 | M | Tongue |
| 20 | T3N0M0 | 45 | M | Buccal Mucosa |
| 21 | T3N1M0 | 60 | F | Tongue |
| 22 | T4aN3bM0 | 43 | M | Buccal Mucosa |
| 23 | T2N2aM0 | 46 | M | Oropharynx |
| 24 | T2N0M0 | 46 | M | Buccal Mucosa |
| 25 | T4aN0M0 | 38 | M | Buccal Mucosa |
| 26 | T3N1M0 | 40 | M | Oropharynx |
| 27 | T4aN1M0 | 76 | M | Lower alveolus |
| 28 | T2NoMo | 47 | M | Tongue |
| 29 | T3N1M0 | 43 | F | Buccal Mucosa |
| 30 | T3N1M0 | 46 | M | Buccal Mucosa |
| 31 | T4bN2aM0 | 66 | M | Oropharynx |
| 32 | T1N0M0 | 50 | M | Buccal Mucosa |
| 33 | T2N1M0 | 68 | M | Oropharynx |
